# Prognostic Significance of Twist and N-Cadherin Expression in NSCLC

**DOI:** 10.1371/journal.pone.0062171

**Published:** 2013-04-23

**Authors:** Linping Hui, Siyang Zhang, Xinjun Dong, Dali Tian, Zeshi Cui, Xueshan Qiu

**Affiliations:** 1 Laboratory Center, The Fourth Affiliated Hospital of China Medical University, Shenyang, China; 2 Center of Laboratory Technology and Experimental Medicine, China Medical University,Shenyang, China; 3 Department of Pathology, The First Affiliated Hospital and College of Basic Medical Sciences of China Medical University, Shenyang, China; 4 Department of Thoracic Surgery, The Fourth Affiliated Hospital of China Medical University, Shenyang, PR China; Roswell Park Cancer Institute, United States of America

## Abstract

**Background:**

Metastasis is the most common cause of disease failure and mortality for non-small cell lung cancer after surgical resection. Twist has been recently identified as a putative oncogene and a key regulator of carcinoma metastasis. N-cadherin is associated with a more aggressive behavior of cell lines and tumors. The aim of this study was to evaluate the clinical relevance of Twist and N-cadherin expression in NSCLC, and the effects of Twist1 knockdown on lung cancer cells.

**Methods:**

We examined the expressions of Twist and N-cadherin by immunohistochemistry in 120 cases of non-small cell lung cancer (including 68 cases with follow-up records). We also analyzed Twist1 and N-cadherin mRNA expression in 30 non-small cell lung cancer tissues using quantitative reverse transcription polymerase chain reaction. The functional roles of Twist1 in lung cancer cell lines were evaluated by small interfering RNA-mediated depletion of the protein followed by analyses of cell apoptosis and invasion.

**Results:**

In lung cancer tissues, the overexpression rate of Twist was 38.3% in lung cancer tissues. Overexpression of N-cadherin was shown in 40.83% of primary tumors. Moreover, Twist1 mRNA expression levels correlated with N-cadherin mRNA levels. Furthermore, overexpression of Twist1 or N-cadherin in primary non-small cell lung cancers was associated with a shorter overall survival (*P*<0.01, *P*<0.01, respectively). Depleting Twist expression inhibited cell invasion and increased apoptosis in lung cancer cell lines.

**Conclusions:**

The overexpression of Twist and N-cadherin could be considered as useful biomarkers for predicting the prognosis of NSCLC. Twist1 could inhibit apoptosis and promote the invasion of lung cancer cells**,** and depletion of Twist1 in lung cancer cells led to inhibition of N-cadherin expression.

## Introduction

Lung cancer is a common malignancy, which causes millions of deaths worldwide every year. Non-small cell lung cancer (NSCLC) comprises approximately 80% of lung cancers, and nearly 50% of patients with stage I NSCLC die within 10 years of diagnosis [1]–[2]. Although major advances in surgical techniques, chemotherapy, radiotherapy and new strategies of treatment, long-term survival is achieved in only 5–10% of NSCLC patients [3]. The failure of NSCLC therapy and poor prognosis of the disease are mostly attributed to the development of local and distant metastases [4]. In this regard, the acquisition of new therapeutic targets that play important roles in pulmonary carcinogenesis, progression and metastasis will be essential for improving therapeutic intervention and prognosis of lung cancers.

Twist, a highly conserved basic helix-loop-helix (bHLH) transcription factor, is characterized by a basic DNA binding domain that targets the consensus E-box sequence 5′-CANNTG-3′ and a helix-loop-helix domain. In mammals, two Twist-like proteins, Twist1 and Twist2, share high structural homology. The N-termini of Twist1 and Twist2 are more divergent, and Twist2 lacks a glycine-rich region that is present in Twist1 [5]. Twist has been recently identified as a putative oncogene and a key regulator of carcinoma metastasis [6]–[8]. Suppression of Twist expression inhibited the ability of 4T1 cells to metastasize from the mammary gland to lung of BALB/c mice [7]. N-cadherin is a transmembrane glycoprotein composed of extracellular domains that mediate homophilic interactions between neighboring cells, predominantly via a peptide domain containing the His-Ala-Val (HAV) amino acid sequence, which is located near the N-terminus [9]–[10]. N-cadherin mediates cell-cell adhesion via homophilic binding and the stability of cadherin-mediated cell adhesion. N-cadherin expression is associated with a more aggressive behavior of cell lines and tumors, such as invasion and migration [11]–[12]. Interestingly, Twist1 overexpression is correlated to abnormal expression of N-cadherin mRNA in human diffuse-type gastric cancer and Twist1 is a transcriptional activator of N-cadherin gene in prostate cancer cells [13]–[14].

In previous studies, we detected Twist over-expression in NSCLC and found that high expression of Twist was associated with differentiation in NSCLC [15]. However, the correlation of Twist and N-cadherin expression in NSCLC has not been elucidated. Therefore, in this study, we explored the relationship between Twist and N-cadherin in 120 cases of NSCLC specimens, and their impacts on lung cancer patients’ outcomes. In addition, the effects of Twist on N-cadherin expression, cell apoptosis and invasion were investigated in lung cancer cell lines using small interfering RNAs.

## Materials and Methods

### Patients and Tissue Samples

120 cases of NSCLC and 20 corresponding nontumorous lung tissues were obtained from the 1st January 2001 to the 31st December 2010, following surgical resection at the First Affiliated Hospital of China Medical University. None of the patients had received radiation therapy or chemotherapy before surgery. Of the patients, 81 were male and 39 were female, with a median age of 60 years (range 20–83 years). Formalin-fixed paraffin-embedded sections of tumor were stained routinely with hematoxylin and eosin, and reviewed by two senior pathologists in order to determine the histological type, according to the WHO classification of lung and pleural tumors (2004). There were 63 cases of squamous cell carcinoma and 57 cases of adenocarcinoma. The TNM taging system of the International Union Against Cancer (7th Edition) was used to classify specimens as stages I (n = 38), II (n = 34), III (n = 47), and IV (n = 1). Lymph node status was determined by routine pathological examination of dissected nodes. Among the 120 cases, 68 cases had complete follow-up records. The survival time was calculated from the operation day to death or until the last follow-up date (December 2010). The following-up of the surviving patients averaged 30.8 months and ranged from 3 to 72 months. In addition, 30 fresh samples including both NSCLC tissues and corresponding nontumorous lung tissues were obtained for the extraction of RNA at the Fourth Affiliated Hospital of China Medical University between 2010 and 2011. Of the 30 patients, 17 were stage I-II and 13 were stage III-IV. All human tissues were obtained in accordance with human subjects protocols approved by the China Medical University Review Board. Tumor and corresponding nontumorous tissues were obtained with written informed consent from adult patients operated for lung tumor.

### Immunohistochemistry

Formalin-fixed paraffin-embedded specimens were cut into 4 µm-thick sequential sections. After dewaxing in xylene and rehydrating stepwise in ethanol, antigen retrieval was carried out using 0.01 mol/L of citrate buffer (pH 6.0) for 2 min in an autoclave. Immunostaining was performed by the streptavidin-peroxidase (S-P) method (Ultrasensitive™ MaiXin, Fuzhou, China). Hydrogen peroxide (3%) was applied to block endogenous peroxidase activity, and normal goat serum was used to reduce nonspecific binding. Then sections were incubated with Twist (H-81) rabbit polyclonal antibody (1∶100 dilution) (Santa cruz, CA, USA) or N-cadherin rabbit monoclonal antibody (1∶150 dilution) (Santa cruz, CA, USA) overnight at 4°C. The peroxidase reaction was developed with DAB (Maixin Biotechnology, Fuzhou, China), and sections were counterstained with hematoxylin, dehydrated through alcohol, and mounted using a standard procedure. All the samples were evaluated by 2 independent pathologists, the scoring criteria was in the light of the scoring system described previously [16]–[17]. The heterogeneity of each tissue section was not very high, and 10 individual fields were randomly selected per slide to compensate for the heterogeneity at 400×magnification. The intensity of Twist or N-cadherin staining was scored as 0 (no signal), 1 (weak), 2 (moderate), and 3 (marked). Scores of positive cells percentage were assigned as 0 (<5%), 1 (5%–25%), 2 (26%–50%), and 3 (≥51%). The scores of each view were multiplied to give a final score of 0–9, and the final score of one sample was the mean of 10 microscopic fields. Tumors were finally determined as negative (−), score 0; lower expression (+), score 1; moderate expression (++), score 2–4; and higher expression (+++), score 6–9. Tumor sample scored (++) to (+++) were considered overexpression, while samples scored (−) to (+) were considered normal expression.

### Cell Culture

A549, LTEP-α-2, H157 and SPC-A1 (adenocarcinoma), LH7 (large carcinoma), LK2 (squamous carcinoma) cell lines and Human Bronchial Epithelial (HBE) from our previous published paper [18]–[19] were maintained in RPMI1640 medium supplemented with 10% fetal bovine serum (Gibco, USA), 100 units/ml streptomycin, and 100 units/ml penicillin in a humidified 5% v/v CO2 atmosphere.

### Gene Knockdown by Small Interfering RNA

Three pairs of the Twist1 siRNA sequence were designed and synthesized by GenePharma (Shanghai, China). Considering relative effectiveness and stability, the following siRNA sequence was selected according to our pilot experiments: 5′-GAU GGC AAG CUG CAG CUA UTT-3′, 5′-AUA GCU GCA GCU UGC CAU CTT-3′; it was transfected into A549 and LTE cells. A non-silencing siRNA sequence was used as a negative control (5′-UUC UCC GAA CGU GUC ACG UTT-3′, 5′-ACG UGA CAC GUU CGG AGA ATT-3′). SiRNA transfections were performed according to the manufacturer’s instructions. For transfections, cells were seeded in a 6-well plate 24 h before the experiment. Briefly, twist1 siRNA or negative control siRNA of 100 pmol was diluted in 250 µl of Opti-MEM I medium. Next, 5 µl Lipofectamine 2000 was diluted in 250 µl of Opti-MEM I Medium. After 5 min incubation, the diluted siRNA was mixed with diluted Lipofectamine 2000 gently and incubated for 20 min at room temperature. The oligomer-lipofectamine complexes were applied to the subconfluent cells. Following transfection, the Twist1 mRNA and protein levels were assessed 48 h later.

### Quantitative Real-time Polymerase Chain Reaction (qRT-PCR)

Total RNA was isolated using RNAiso Plus (Takara). Quantitative reverse transcriptase PCR (qRTPCR) was performed using the reverse transcriptase kit from Takara (*PrimeScript*® RT reagent Kit-Perfect Real Time). Primers were designed using the Primer Express software. The primers used were as follows: Twist1-forward, 5′-GGC ACC ATC CTC ACA CCT CT-3′; Twist1-reverse, 5′-GCT GAT TGG CAC GAC CTC T-3′; N-cadherin-forward, 5′-ATT GGA CCA TCA CTC GGC TTA-3′; N-cadherin-reverse, 5′-CAC ACT GGC AAA CCT TCA CG-3′; GAPDH-forward, 5′-GCA CCG TCA AGG CTG AGA AC-3′; GAPDH-reverse, 5′-TGG TGA AGA CGC CAG TGG A-3′. Quantitative realtime polymerase chain reaction (QPCR) was done using the SYBR Green PCR Master Mix from Takara (*Premix Ex Taq*™-Perfect Real Time) in a total volume of 20 µl on the 7300 Real-Time PCR System (Applied Biosystems): 95°C for 30 s, 50 cycles of 95°C for 5 s, 60°C for 31s. GAPDH was used as the reference gene. Experiments were repeated in triplicates. The relative levels of gene expression were represented as ΔCt = Ct_target gene_−Ct_reference gene_), and the fold differences of gene expression between NSCLC tissues and corresponding nontumorous lung tissues were calculated by the 2^−ΔΔCt^ method.

### Western Blot

Cells were lysed in lysis buffer: 50 mM Tris-HCl (pH 8.0), 150 mM NaCl, 0.5% Nonidet P40, 0.5% sodium deoxycholate, and phenylmethylsulfonyl fluoride (PMSF, all from Sigma). The protein concentration was determined by the bicinchoninic acid (BCA) assay (Beyotime, China). Each sample (60 µg) was separated by 8% or 10% SDS-PAGE and transferred to a polyvinylidene fluoride membrane (Millipore, Billerica, MA, USA). After blocking with 5% BSA in Tris-buffered saline-Tween 20 (TBST; 20 mM Tris–HCl, 500 mM NaCl, 0.05% Tween-20), membranes were incubated with primary antibodies for Twist (1∶500), N-cadherin (1∶500) or β-actin (1∶2000; Santa Cruz) overnight at 4°C. After incubation with peroxidase-coupled anti-mouse-IgG or anti-rabbit-IgG (SABC, Beijing, China) at 37°C for 2 h, the protein bands were visualized by enhanced chemiluminescence reagent (SuperSignal West Pico; Thermo Scientific) in the MF-ChemiBIS 3.2 (DNR BioImaging system, Israel). The optical density of each protein band was measured using the Image Quant software. The ratio between the optical density of interest protein and β-actin of the same sample was calculated as the relative content of the protein detected.

### Immunofluorescence Staining

Cells were fixed with 4% paraformaldehyde and rinsed in PBS. Cells were blocked with 5% BSA at 37°C for 60 min. The primary rabbit polyclonal antibody for Twist or N-cadherin (1∶100) was then added to cells and incubated at 4°C overnight, which was followed by the incubation with secondary antibodies conjugated to TRITC-labelled. The nuclei were counterstained with DAPI at 37°C for 20 min. Fluorescent images were recorded in the same conditions using Confocal Laser Scanning Microscope (FV1000, Olympus, Japan).

### Cell Apoptosis Assay

Cell apoptosis was examined by flow cytometry using an Annexin V-FITC apoptosis detection kit (KeyGen, Nanjing, China), following the manufacturer’s protocol. Cells were washed twice in ice-cold PBS and resuspended in 1×binding buffer (1×10^6^/ml). Cells of 100 µl (1×10^5^) were gently mixed with 5 µl Annexin V-FITC and 5 µl PI, and incubated for 15min at room temperature away from light. After another 400 µl 1×binding buffer was supplemented, cell apoptosis was detected in flow cytometer (FACSCalibur, BD Biosciences, USA). Results are representative of three individual experiments.

### Cell Invasion Assay

The cell invasion assay was performed using a 24-well Transwell chamber (Costar, Corning, USA). 48 h after transfection, A549 or LTE cells were trypsinized and transferred to the upper chamber with a 8 µm pore size insert precoated with Matrigel (BD Biosciences, USA) and cultured in serum-free medium for 24 h. Medium supplemented with 10% FBS was added to the lower chamber as the chemoattractant. The numbers of invaded cells were counted in 10 randomly selected fields at a high-magnification (400×). The experiments were performed in triplicates.

### Statistical Analysis

The SPSS 13.0 software was applied to complete data processing. χ2-test and non-parametric test were applied to analyze the correlations between Twist or N-cadherin expression and clinicopathological characteristics. The association between Twist and N-cadherin immunointensity of the same specimens was analyzed using Spearman rank correlation test. Kaplan–Meier curves were used for survival analysis, and log-rank was determined based on the differences. A multivariate analysis was performed using the Cox regression model to study the effects of different variables on survival. One-way ANOVA, independent T test and Kruskal–Wallis were selected according to the normality of the data.T-test or One-way ANOVA was used to compare the differences between cells with various treatments. All data were represented as mean±SD and results were considered statistically significant when the *p*-value was less than 0.05.

## Results

### Twist Expression and Clinicopathological Parameters

**Figure 1:**
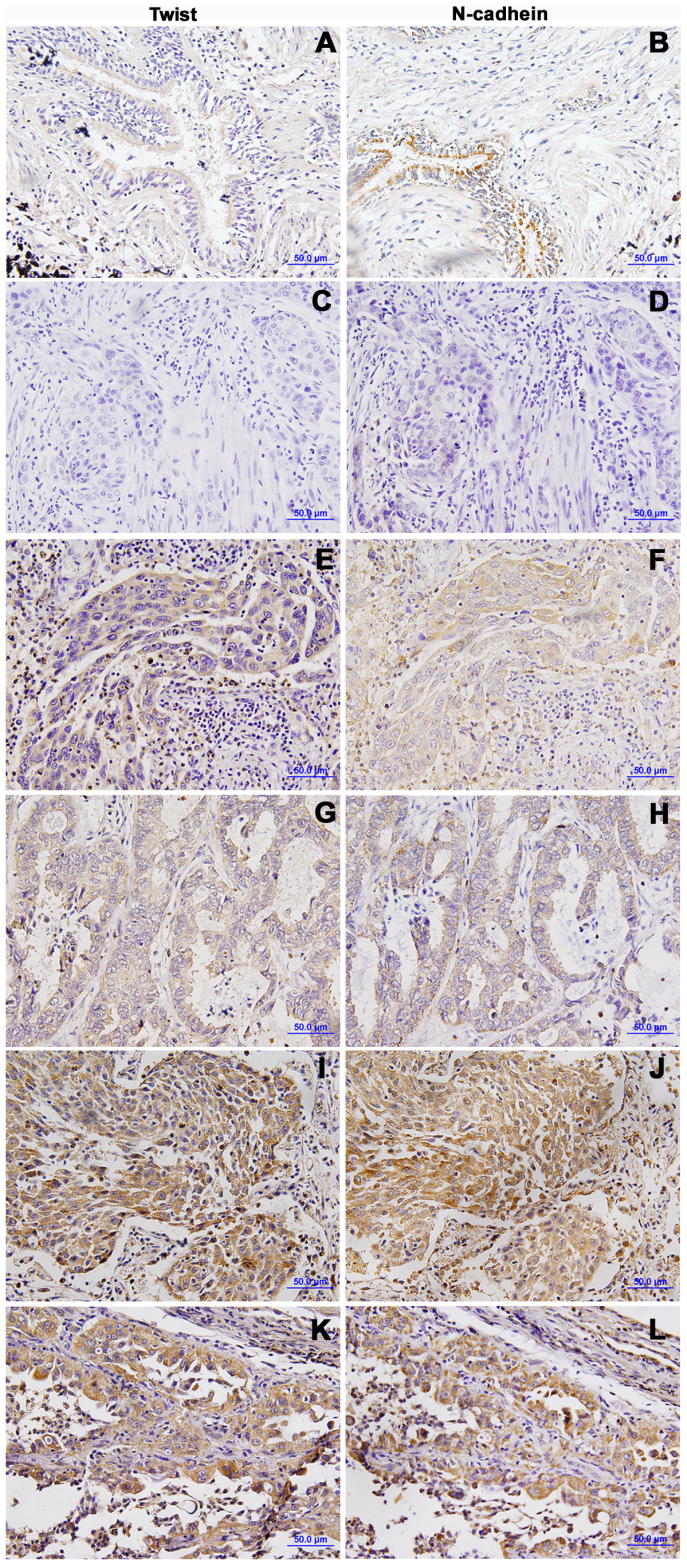
Immunohistochemical staining of Twist and N-cadherin in nontumorous tissues and NSCLCs. (A, B) Twist and N-cadherin were weakly expressed in bronchial epithelium; (C, D) NSCLC −; (E, F) squamous cell carcinoma +; (G, H) adenocarcinoma +; (I, J) squamous cell carcinoma ++; (K, L) adenocarcinoma ++.

Twist was mainly found in cytoplasm by immunohistochemical staining. In lung cancer tissues, the overexpression rate of Twist was 38.3% (46 of 120), which was significantly higher than that in corresponding nontumorous lung tissues (*P*<0.01; Fig. 1). The relationship between Twist expression and different clinicopathological factors was shown in Table 1. The overexpression of Twist in tumors of stage III–IV (56.25%) was higher than that in stage I (21.05%) and II (32.35%, *P*<0.01); the overexpression rate of Twist in poor differentiated cancer tissues (66.67%) was higher than that in well (20.00%) and moderate (26.79%) differentiated cancer tissues (*P*<0.01) and it was also higher in cases with lymphatic metastasis than in cases without lymphatic metastasis (48.58% versus 24.0%, *P*<0.01). We also examined the expression of N-cadherin in serial sections (Figure 1). N-cadherin overexpression rate was 40.83% (49 of 120). The overexpression of N-cadherin in stages III–IV (39.58%) was higher than that in stages I (31.58%) and II (23.53%, *P*<0.01); N-cadherin expression in poor differentiated (64.10%) cancer tissues was higher than that in well and moderate differentiated cancer tissues (16.0% and 35.71%, *P*<0.01). In addition, a multivariate analysis demonstrated that N-cadherin overexpression and Twist/N-cadherin co-overexpression were significant predictors of survival (Table 2). However, overexpression of Twist was not an independent prognostic factor by a multivariate Cox regression analysis (p = 0.308), but the above data are sufficient to show a correlation between Twist expression and poor prognosis of NSCLC patients. A significant correlation was found between Twist and N-cadherin expressions by Spearman correlation analysis (R = 0.565, *P* = 0.000, Table 3).

**Table 1:** Relationships between Twist, N-cadherin expression and clinicopathological parameters in 120 cases of NSCLC patients.

Features	Cases(n)	Twist normal expression	Twist overexpression	*P*	N-cadherinnormal expression	N-cadherin overexpression	*P*
Sex				0.436			0.248
Male	81	48	33		45	36	
Female	39	26	13		26	13	
Age				0.523			0.724
<60 years	54	35	19		31	23	
≥60years	66	39	27		40	26	
TNM stage				0.003			0.001
I	38	30	8		26	12	
II	34	23	11		26	8	
III–IV	48	21	27		19	29	
Differentiation				0.000			0.000
Well	25	20	5		21	4	
Moderate	56	41	15		36	20	
Poor	39	13	26		14	25	
Histology type				0.750			0.113
SCC	63	38	25		33	30	
AC	57	36	21		38	19	
Nodal status				0.007			0.098
N0	50	38	12		34	16	
N1 N2 N3	70	36	34		37	33	

**Table 2:** Correlation between Twist, N-cadherin expression by Pearson correlation analysis in NSCLC patients.

	N-cadherin overexpression	N-cadherin normal expression	Total
Twist overexpression	35	11	46
Twist normal expression	14	60	74
Total	49	71	120

**Table 3:** Multivariate analyses for overall survivals of 68 NSCLC patients.

Variables	Wald	Exp(B)	95.0% CI for Exp(B)	*P*
sex	0.961	0.695	0.336–1.439	0.327
age	2.143	1.533	0.865–2.714	0.143
Histology type	3.431	1.777	0.967–3.264	0.064
Lymph node metastasis	0.190	1.279	0.423–3.872	0.663
TNM Stage				
I	5.490			0.064
II	4.341	0.506	0.266–0.960	0.037*
III–IV	3.596	0.540	0.286–1.021	0.058
Differentiation				
Well	2.571			0.276
Moderate	1.519	0.618	0.287–1.329	0.218
Poor	2.375	0.587	0.298–1.156	0.123
Twist overexpression	1.038	1.463	0.704–3.039	0.308
N-cadherin overexpression	4.860	2.386	1.101–5.170	0.027*
Twist/N-cadherin co-overexpression				
0–1				
2	14.265	3.704	1.877–7.306	<0.001

0, normal expression; 1, one overexpression; 2 co-overexpression of two markers.

* *P*<0.05.

**Figure 2:**
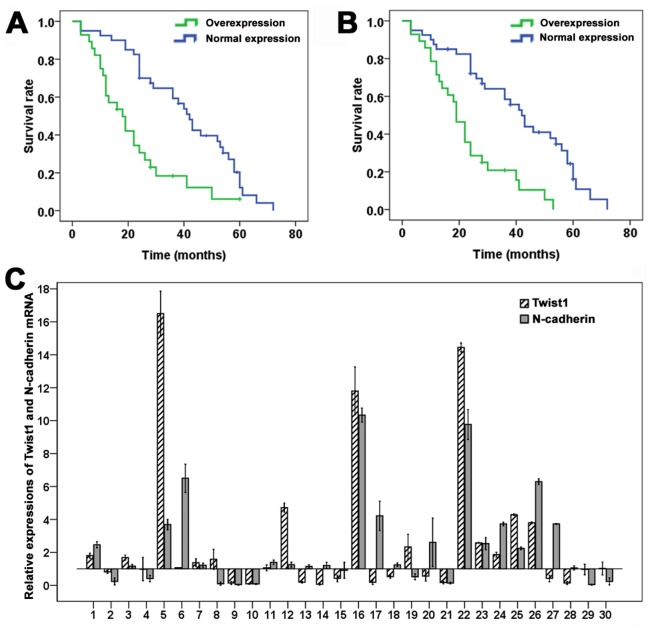
Kaplan–Meier survival curves of NSCLC patients. (A) Stratified according to Twist expression (log-rank test, *P*<0.01). (B) Stratified according to N-cadherin expression (log-rank test, *P*<0.01). (C) The fold differences of Twist1 and N-cadherin mRNA between NSCLC tissues and corresponding nontumorous lung tissues were calculated and expressed graphically. The results were representative of three independent experiments.

In order to verify the results of immunohistochemistry, we detected the mRNA expressions of Twist1 and N-cadherin by qRT-PCR in 30 cases of lung cancer and paired nontumorous tissues. Of the 30 patients, 17 (56.7%) showed a higher level of Twist1 mRNA in lung cancer specimens than in nontumorous specimens and 20 (66.7%) showed a higher level of N-cadherin mRNA in cancer specimens than in nontumorous specimens (Fig. 2C). Kruskal–Wallis test was used for the comparison of Twist1 and N-cadherin mRNA levels in early(I-II) and advanced(III-IV) stage tumors. The overexpression of Twist1 mRNA were associated with TNM stage (Chi-Square = 9.984, *P*<0.01). And the overexpression of N-cadherin mRNA were associated with TNM stage (Chi-Square = 14.557, *P*<0.01). Moreover, the Twist1 mRNA expression was associated with N-cadherin mRNA expression (R = 0.706, *P* = 0.000).

### Expression of Twist and N-cadherin was Associated with Poor Prognosis of NSCLC Patients

To evaluate whether the expressions of Twist and N-cadherin in primary non-small cell lung cancer were related to patient’s outcome, Kaplan-Meier survival curves were constructed. A significant correlation between the immunointensity of Twist and the overall survival of patients was shown (log-rank test, *P*<0.001). The overall survival was significantly lower in patients with Twist-overexpressed tumors than in patients with Twist-normal expression (*P*<0.01; Fig. 2A). In addition, the overall survival was also significantly lower in patients with N-cadherin overexpressed tumors than in patients with N-cadherin normal expression (*P*<0.01; Fig. 2B).

### Twist Expression in HBE and 5 Lung Cancer Cell Lines

**Figure 3:**
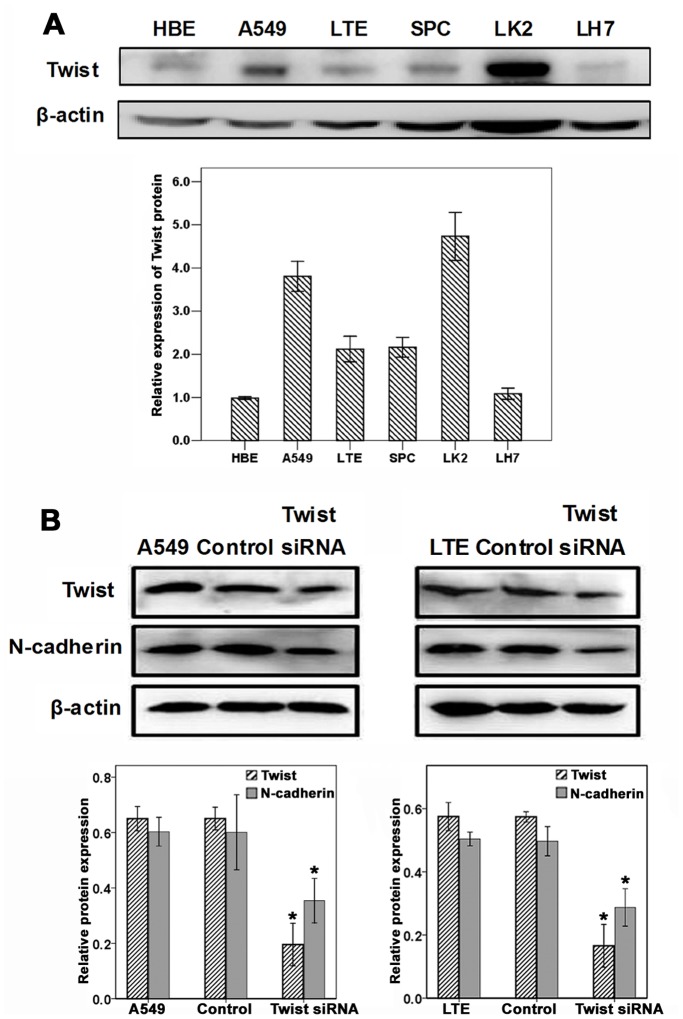
Suppression of Twist1 Expression in A549 and LTE cells downregulated the expression of N-cadheirn. (A) Different levels of Twist protein were detected in HBE, adenocarcinoma cells of A549, LTE and SPC, squamous cell carcinoma of LK2, and large cell carcinoma of LH7 by western blot assays. (B) The expressions of Twist and N-cadherin were decreased in A549 and LTE cells transfected with Twist siRNA, compared with the control cells. bar, SD; *P<0.01.

Relative expression level of Twist was analyzed by western blot in a panel of lung cancer cell lines (Fig. 3A). To determine whether Twist is required for apoptosis and invasion of lung cancer cells, siRNA was employed to knock down Twist1 expression in A549 and LTE cells, since they had higher level of Twist compared with human bronchial epithelial cells of HBE. The Twist1 expression was unaffected in cells transient transfected with nonsilencing control siRNA, whereas Twist1-siRNA considerably reduced the protein level 48 h after siRNA transfection (*P*<0.01). As expected, the N-cadherin downregulation was also observed in both cell lines transfected with Twist1 siRNA by western blot assays (Fig. 3B).

**Figure 4:**
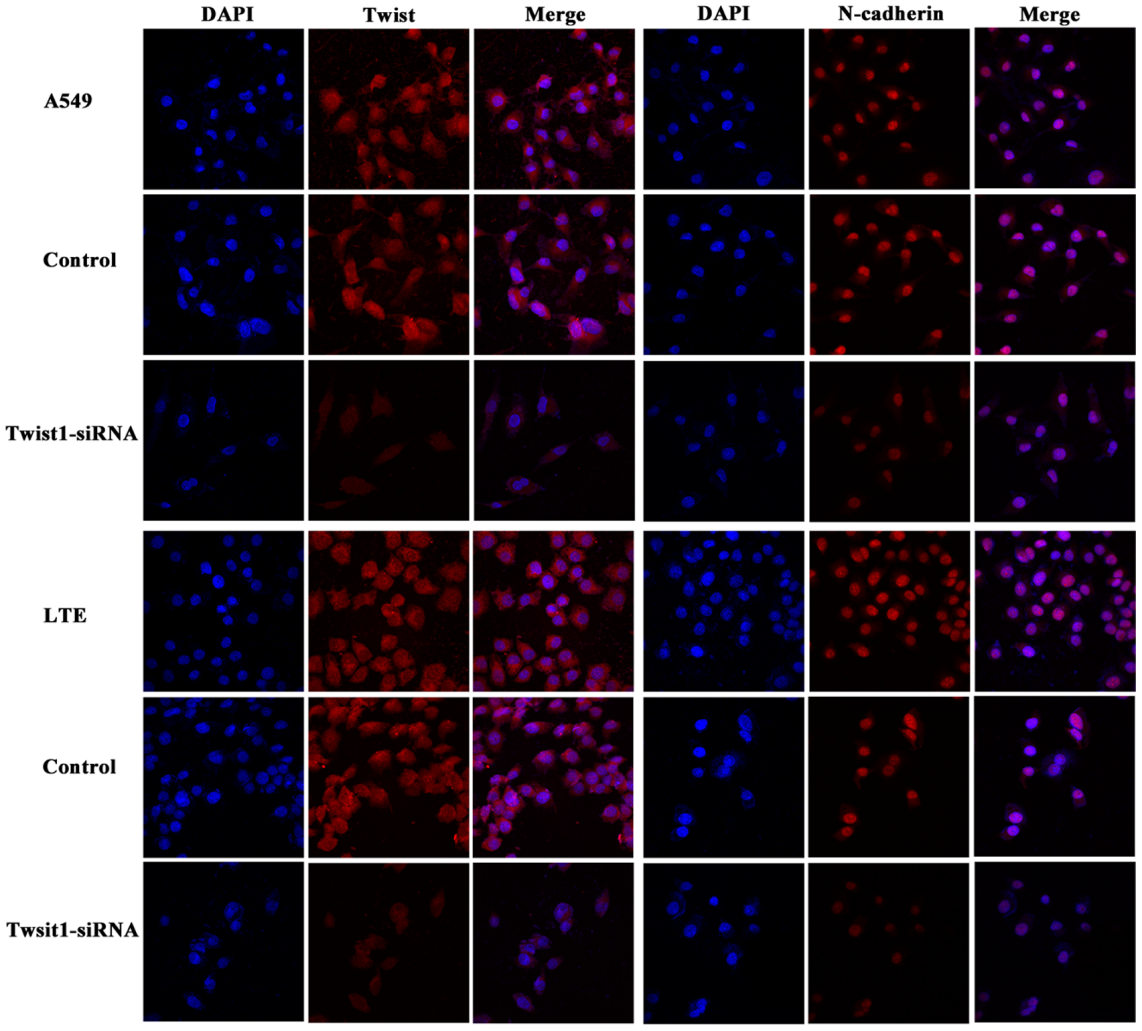
Immunofluorescent staining of Twist and N-cadherin in A549 and LTE cells transfected with Twist siRNA (600×magnification).

Immunofluorescent staining was used to detect the expressions of Twist and N-cadherin in A549 and LTE cells. The fluorescence signal of Twist and N-cadherin protein was conspicuous in A549 and LTE cells. Furthermore, N-cadherin was observed downregulated in Twist knock-down cells (Fig. 4), which was coincident with the results of western blot assays.

### Depletion of Twist1 Inducing Apoptosis of Lung Cancer Cells

**Figure 5:**
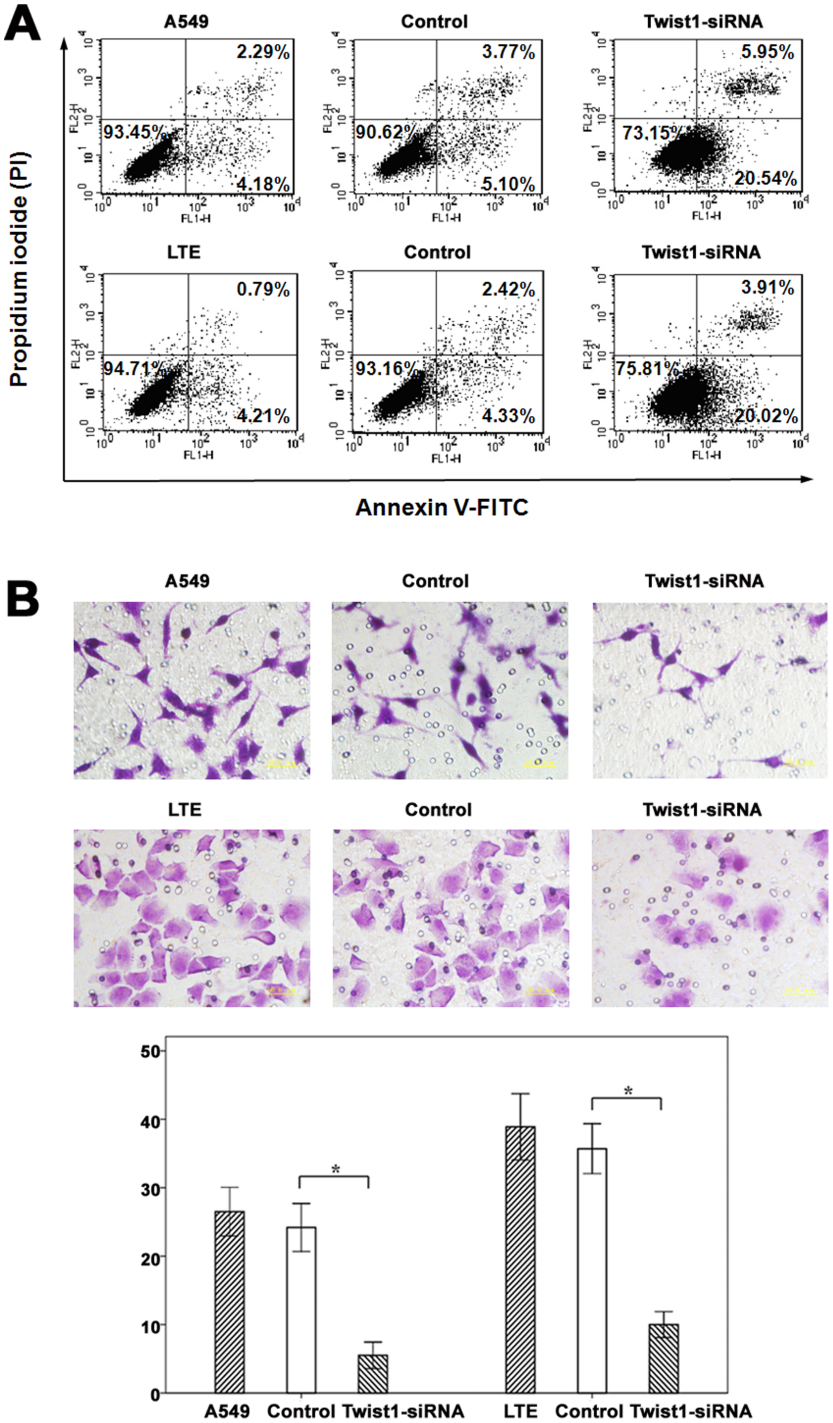
Interruption of Twist expression promoted cell apoptosis. (A) The apoptotic rates of Twist knockdown cells were moderately increased in contrast to control siRNA transfected and untreated cells. Apoptotic cells were identified by double supravital staining with FITC conjugated Annexin-V and PI. Upper right, late apoptotic cells; lower left, fully viable cells; lower right, early apoptotic cells; upper left, necrotic cells. (B) Depletion of Twist inhibited the invasive ability of lung cancer cells. Cell invasion was examined using cell culture chambers with Matrigel. The representative microscopic fields of invasive cells transfected with Twist and control siRNA, and untreated cells were shown, respectively. The histogram showed that the number of invasive cells (A549 and LTE) transfected with Twist siRNA was significantly fewer (*P*<0.05) than cells transfected with control siRNA or untreated. Columns, mean (n = 10); bar, SD; *P<0.01.

Flow cytometry was used to detect cell apoptosis. The flow cytometry results showed a moderate increase in apoptosis after A549 and LTE cells transfected with Twist1 siRNA (approximately 27.23±1.27% and 25.12±1.03% respectively, P<0.05), compared with the cells transfected with empty vector (approximately 8.11±0.45% and 6.67±0.17% respectively) or untreated (approximately 6.82±0.83% and 5.17±0.22% respectively, Fig. 5A).

### Suppression of Twist1 Expression Inhibited the Invasion of Lung Cancer Cells

We further examined whether cell invasive capacity was altered in Twist1 depleted cells. Invasion assays showed that both A549 and LTE cells transfected with Twist siRNA had fewer cells invaded (5.50±0.97 and 10.00±0.94 respectively, P<0.01) than the cells transfected with control siRNA (24.20±1.75 and 35.70±1.83 respectively) or untreated (26.50±1.78 and 38.90±2.42 respectively, Fig. 5B).

## Discussion

Transcription factor Twist plays an important role in embryogenesis and tumorigenesis, and also regulates epithelial-mesenchymal transition in the development of metastatic cancer cells. Previous studies have demonstrated that Twist is overexpressed in several cancers and involved in tumor progression [20]–[26]. Twist expression is found elevated in gastric cancer, hepatocellular carcinima, NSCLC and was correlated with clinicopathological parameters [27]–[31]. N-cadherin enhances cell motility and is involved in migration, invasion of various tumor cells and N-cadherin is a prognostic marker of progression in superficial urothelial tumors [32]. Notably, Twist is known as an activator of N-cadherin in *Drosophila*, and up-regulation of Twist stimulated N-cadherin expression in MKN28 cells with TwistS transfection [33]–[34]. However, the expressions of Twist and N-cadherin in NSCLC as well as their correlations with clinical and pathological factors have not been evaluated.

We previously demonstrated that Twist expression was significantly higher in NSCLC, which was associated with differentiation of NSCLC [15]. In this study, we further examined the expressions of Twist and N-cadherin in 120 NSCLC specimens. We found that the overexpression of Twist was correlated with advanced TNM stage, poor differentiation, lymph node metastasis and a shorter survival rate of patients, which were consistent with previous studies in primary NSCLC [28]–[30]. Moreover, we noticed that the overexpression of N-cadherin was significantly associated with advanced TNM stage, poor differentiation and reduced overall survival of lung cancer patients. We also found that the higher expression of Twist was associated well with the higher expression of N-cadherin at both protein and mRNA levels. Moreover, expressions of Twist and N-cadherin protein were significantly associated with reduced overall survival for non-small cell lung cancer patients. Taken together, Twist and N-cadherin are probably oncogenic proteins in NSCLC and may serve as predictors of survival in lung cancer patients.

Besides NSCLC specimens, the expression of Twist was further investigated in HBE and 5 lung cancer cell lines. Our results showed that Twist expression was higher in lung cancer cells than in HBE cells. Moreover, we found that Twist1 knockdown caused decreased N-cadherin expression in both A549 and LTE cells. The correlation between Twist and N-cadherin expressions was also confirmed in cell lines, which was consistent with the data from NSCLC tissues. To investigate the potential function of Twist in cell apoptosis and invasion, we silenced Twist1 in A549 and LTE cells. We found that apoptosis was significantly induced in Twist1-siRNA transfected cells. Previous studies have revealed that Twist inhibits apoptosis in other human cancer cells [35]. Although several signaling pathways such as ARF/MDM2/p53, TNF-a and IGF have been reported to be associated with the anti-apoptotic effects of Twist in osteoblasts and fibroblasts [36]–[37], signalings involved in the Twist-associated anti-apoptotic role of lung cancer cells required further studies. A recent study indicated that Twist increased MMP9 activity in lung cancer H358 cells [38]. In this study, cell invasion assays showed that Twist1 knockdown blocked the invasion of lung cancer A549 and LTE cells, suggesting that Twist1 had an important effect on cell invasion.

In conclusion, our results showed a close correlation between the expression of Twist and N-cadherin in NSCLC. The overexpressions of Twist/N-cadherin could be considered as useful biomarkers for predicting the prognosis of NSCLC. Depletion of Twist1 induced apoptosis, inhibited invasion and led to the reduction of N-cadherin expression in lung cancer cells. This study thus provides further impetus for exploiting Twist/N-cadherin associated pathway as a potential target for the treatment of NSCLC.
